# Jumping performance based on duration of rehabilitation in female football players after anterior cruciate ligament reconstruction

**DOI:** 10.1007/s00167-018-5154-5

**Published:** 2018-09-28

**Authors:** Amelia J. H. Arundale, Joanna Kvist, Martin Hägglund, Anne Fältström

**Affiliations:** 1 Division of Physiotherapy, Department of Medical and Health Sciences, Linköping University, Linköping, Sweden; 2 Division of Physiotherapy, Department of Neurobiology, Care Sciences and Society, Karolinska Institutet, Stockholm, Sweden; 3 Football Research Group, Linköping University, Linköping, Sweden; 4 Region Jönköping County, Rehabilitation Centre, Ryhov County Hospital, Jönköping, 551 85 Sweden

**Keywords:** ACL injury, Valgus, Soccer

## Abstract

**Purpose:**

To determine if female football players who had longer durations of rehabilitation, measured in months, after anterior cruciate ligament reconstruction would have lower tuck jump scores (fewer technique flaws) and smaller asymmetries during drop vertical jump landing.

**Methods:**

One-hundred-and-seventeen female football players, aged 16–25 years, after primary unilateral ACL reconstruction (median 16 months, range 6–39) were included. Athletes reported the duration of rehabilitation they performed after anterior cruciate ligament reconstruction. Athletes also performed the tuck jump and drop vertical jump tests. Outcome variables were: tuck jump score, frontal plane knee motion and probability of peak knee abduction moment during drop vertical jump landing.

**Results:**

There was no difference in tuck jump score based on duration of rehabilitation (n.s.). No interaction (n.s.), difference between limbs (n.s.), or duration of rehabilitation (n.s.) was found for peak knee abduction moment during drop vertical jump landing. No interaction (n.s.) or difference between limbs (n.s.) was found for frontal plane knee motion, but there was a difference based on duration of rehabilitation (*P* = 0.01). Athletes with > 9 months of rehabilitation had more frontal plane knee motion (medial knee displacement) than athletes with < 6 months (*P* = 0.01) or 6–9 months (*P* = 0.03).

**Conclusion:**

As there was no difference in tuck jump score or peak knee abduction moment based on duration of rehabilitation, the results of this study press upon clinicians the importance of using objective measures to progress rehabilitation and clear athletes for return to sport, rather than time alone.

**Level of evidence:**

II.

## Introduction

Time from surgery or duration of rehabilitation is an easy measure for athletes, clinicians, and surgeons to track, but time alone may not give the most complete information on an athlete’s recovery [3]. Time is the most common measure for readiness to return to sport [4, 29] but time in rehabilitation does not take into account an athlete’s function, their psychosocial situation, attendance or compliance with rehabilitation and home exercises.

Many athletes expect to return to sport in 6–12 months after ACL reconstruction [2, 32]. However, 12 months after surgery athletes may still have quadriceps strength and single-legged hop asymmetries [5, 7, 24] or may not have returned to their preinjury level of sport [1, 2]. Although asymmetries may not be present in all athletes [10], those athletes who have returned to sport are at a high risk for a second ACL injury [27, 28]. Recently, a community-level study demonstrated a relationship between longer durations of post-operative rehabilitation (≥ 6 months) and greater quadriceps and single-legged hop limb symmetry [7]. Such results support an idea that longer durations of rehabilitation may be related to better outcomes. An idea reinforced by reports that longer time from surgery to return to sport may be related to a lower risk of knee reinjury [11]. However, not all studies have found the same relationship between time from surgery and reinjury risk [16, 31]. Further, as the previous community-level study only examined duration of rehabilitation with strength and single-legged hopping outcomes [7], it is not clear that the same relationship extends to higher level, more demanding jump landings.

The tuck jump test aims to identify technique flaws during plyometric activity, with the goal of identifying athletes that could be at risk for injury [13, 20]. Originally proposed for use in primary ACL injury prevention, both the tuck jump and drop vertical jump (DVJ) tests are clinically feasible tests suggested for assessing athletes’ progression through later stages of rehabilitation or readiness to return to sport after ACL reconstruction [12, 13, 18–21, 23]. Both tests focus on how an athlete lands, particularly observing if the knees collapse medially, a movement pattern thought to be negative [14, 15]. The tuck jump and DVJ tests are quick, require only a small amount of space, and can be recorded using two-dimensional video cameras, allowing clinicians to easily and inexpensively examine higher level sport-related tasks.

The purpose of this study was to explore if there were differences in tuck jump score and asymmetry during DVJ landing based on duration of rehabilitation in female football players. Expanding on the previous findings relating longer durations of rehabilitation to more symmetrical performance [7], the authors hypothesized that female football players who had longer durations of rehabilitation would have lower tuck jump scores (fewer technique flaws) and smaller asymmetries during landing of a DVJ.

## Materials and methods

This study was a secondary aim and analysis of baseline data collected as part of a prospective cohort study. The primary analysis, and its methodology, exploring knee function and return to sport outcomes has been published [10], and prospective results on subsequent injuries are forthcoming. After the primary analysis was published, 40 additional athletes, meeting the same inclusion criteria, were added to the original cohort of 77 athletes [10]. Athletes received written and verbal information about the study, and gave written informed consent prior to testing. One experienced researcher/physiotherapist (AF) collected all data.

Athletes were identified through the Swedish National Knee Ligament Register, and via advertisement on the websites of three regional football districts near Linköping University, Sweden. Inclusion criteria were female football players, 16–25 years old, who had a primary ACL reconstruction in the past 6–36 months at a clinic in one of the three regional football districts. Exclusion criteria were a concomitant posterior cruciate ligament injury, medial or lateral collateral ligament knee injury that was surgically treated [9, 10], never being an active football player, or not returning to football after reconstruction (self-report) [9].

**Fig. 1:**
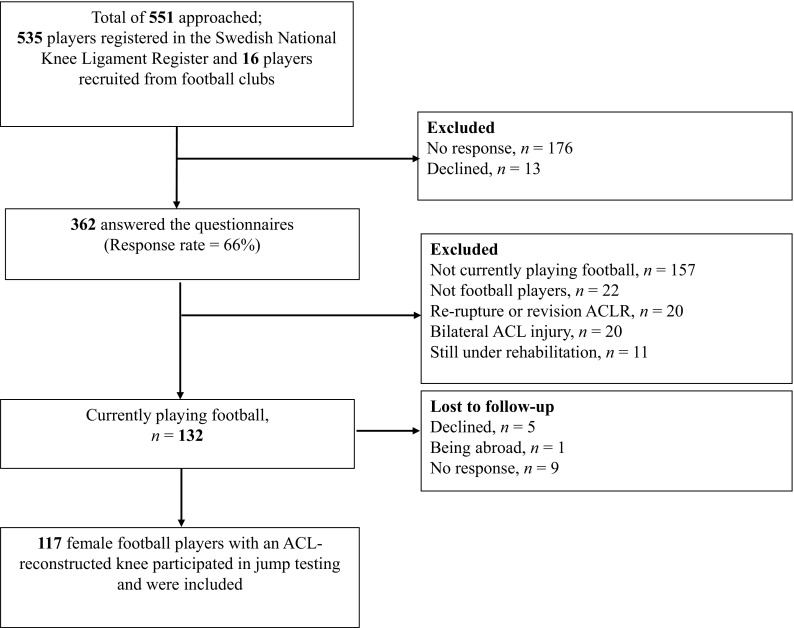
Study flowchart

Five hundred and thirty-five athletes were identified through the Swedish National Knee Ligament Register as meeting inclusion criteria (Fig. 1). These athletes were contacted via mail with information about the study and login details for a web-based questionnaire. In addition, 16 athletes after ACL reconstruction who were not in the Swedish National Knee Ligament Register responded to advertisements (Fig. 1). Of these athletes, 362 answered the web-based questionnaire and 117 met all inclusion criteria and participated in the jump testing.

The web-based questionnaire asked athletes about their age, level of football, and duration of supervised rehabilitation after ACL reconstruction [9]. Duration of rehabilitation after ACL reconstruction was reported as one of the four categories (< 3 months, 3–6 months, 6–9 months, ≥ 9 months). Although it is not medically advised, three athletes had no physiotherapy contact, and two athletes had < 3 months of rehabilitation. For analysis in the present study, those with no rehabilitation, < 3-month, and 3–6-months of rehabilitation were combined into a < 6month group.

Athletes performed one trial of the tuck jump test. The tuck jump involves the athlete jumping continuously for 10 s. The standardized instructions were to lift the knees to hip height and attempt to land in the same place. Athletes were unaware of the grading criteria for the tuck jump test. Two video cameras (Panasonic HC-V500M), one in the frontal and one in the sagittal plane, 5 m and 3.5 m from the athlete, respectively, were used. Video was recorded at 50 Hz with advanced video coding high-definition format at 1080/50p. The tuck jump was analyzed by one researcher (IM), blinded to duration of rehabilitation, according to a clinician-friendly grading tool [13]. Previous research has reported good to excellent reliability using this tool [13, 17, 30]. The grading tool consists of ten  technique flaws grouped into three areas: knee and thigh motion, foot position during landing, and plyometric technique. Technique flaws included; thighs not equal side-to-side, lower extremity valgus at landing, foot placement not parallel, pause between jumps, techniques declining during the 10 s. The tuck jump score ranges from 0 to 10, with zero indicating no flaws and ten being flaws in every criterion. Previously, scores of six or more have been considered abnormal [10, 20]. Athletes were unaware of the grading criteria for the tuck jump test.

To perform the DVJ test athletes stood on a box (31 cm high) with their feet on marks 35 cm apart. Athletes were given standardized instructions to drop down off the box and immediately jump as high as possible reaching with both arms towards a ball suspended at a height of 260 cm. Data were captured with the same two video cameras, 70 cm high, one 3.5 m from the expected landing position in the frontal plane and one 2.5 m in sagittal plane [10]. The three jumps were assessed from the films (IM). Jumps were evaluated on quality in the frontal plane, including symmetry during take-off and landing from the box, knee motion, feet position at landing, and weight displacement. Athletes were unaware of the criteria the jumps were graded on. In keeping with previous analyses [10], the worst assessed jump of the three trials, summarized from all criteria, was used in the analysis [10]. Frontal plane knee motion (medial/valgus or lateral/varus knee displacement) was measured with motion analysis software Dartfish ProSuite (Dartfish Ltd, Fribourg, Switzerland) [8, 25], to two decimal places, in cm as the frontal plane displacement of the knee from initial contact to the end of the deceleration phase of the DVJ. Positive values represented medial/valgus motion. The knee flexion range of motion (°) of the knee closest to the sagittal plane camera was also measured from initial contact to the end of the deceleration phase of the DVJ. To simplify the measurement, the greater trochanter, the lateral knee joint line, the head of the fibula, lateral malleolus, patella tendon, and centre of the patella were marked with a marker pen. Frontal plane knee motion and flexion angle were used in a nomogram to calculate the probability of high knee abduction moment (pKAM) [21, 22]. The range of pKAM was 0–1, which equates to 0 (lowest)–100% (highest probability). The nomogram was based on the player’s weight, tibia length, knee motion in the frontal plane, and knee flexion range of motion and a surrogate value for hamstring–quadriceps ratio (multiplying the player’s mass by 0.01 and adding the resultant value to 1.10) [19, 21, 22].

The study was approved by the Regional Ethical Review Board (Dnr 2012/24-31 and 2013/75-32) and the Swedish National Knee Ligament Register Board.

### Statistical analysis

Variables of interest were the duration of rehabilitation each athlete had (< 6 months, 6–9 months, > 9 months), the total tuck jump score, the frontal plane knee motion and the pKAM during the DVJ. Presence of a concomitant meniscal or cartilage pathology was used as a covariate because such pathologies, particularly if surgically treated or repaired, can prolong rehabilitation.

One-way ANOVAs and chi-squared tests were used to assess if there were differences in demographic and surgical variables between each duration of rehabilitation. An ANCOVA with planned least squares comparisons was used to determine if there were differences between each duration of rehabilitation and total tuck jump score. A chi-square test was used to determine if there were differences in the proportion of athletes who had an abnormal tuck jump score (total tuck jump score ≥ 6) based on duration of rehabilitation. Repeated measures (limb × duration of rehabilitation) ANCOVAs with planned least squares comparisons were used to examine differences in frontal plane knee motion and pKAM between the surgical and non-surgical limbs and between each level of rehabilitation duration. Planned comparisons were the duration of rehabilitation and explored if the main effect was *P* < 0.10. The presence of concomitant meniscal or cartilage pathology was included in all models as a covariate.

Alpha was set a priori at *P* ≤ 0.05 and effect size was reported as partial eta-squared (*np*^2^). The sample size was set by the wider prospective cohort study. Sensitivity analysis indicated that given this sample size (*N* = 117), with a power of 0.80, three groups (duration of rehabilitation) and two measures (limb), an effect size of *np*^2^ = 0.08 could be detected in frontal plane knee motion. Effect sizes were considered small (*np*^2^ = 0.01), medium (*np*^2^ = 0.06), and large (*np*^2^ = 0.14) [6].

## Results

Demographic, anthropometric, surgical and rehabilitation duration variables for the entire group are presented in Table 1. There were no differences in any demographic or surgical variables based on duration of rehabilitation (Table 1).

**Table 1:** Demographic, anthropometric, surgical, and rehabilitation duration variables

Variables	All athletes (*N* = 117)	Duration of rehabilitation			*P* value
		< 6 months (*N* = 27)	6–9 months (*N* = 38)	> 9 months (*N* = 52)	
Age (years)	19.9 ± 2.5	19.6 ± 2.0	19.8 ± 2.5	20.1 ± 2.7	n.s.
Height (cm)	167.9 ± 5.3	167.9 ± 4.7	167.7 ± 5.9	168.0 ± 5.1	n.s.
Weight (kg)	64.9 ± 8.4	64.5 ± 7.4	64.8 ± 9.1	65.1 ± 8.4	n.s.
Graft type (autografts)					
Hamstring	114 (96)	26 (96)	38 (100)	50 (96)	n.s.
Bone–patellar tendon–bone	2 (2)	0 (0)	0 (0)	2 (4)	
Quadriceps tendon	1 (1)	1 (4)	0 (0)	0 (0)	
Concomitant pathology					
Meniscal injury^a^	49 (42)	12 (44)	17 (45)	20 (38)	n.s.
Articular cartilage injury	11 (9)	5 (19)	4 (11)	2 (4)	
Level of football					
Elite	14 (12)	1 (4)	6 (16)	7 (13)	n.s.
Sub-elite	91 (78)	21 (78)	30 (79)	40 (77)	
Recreational	12 (10)	5 (18)	2 (5)	5 (10)	
Months from injury to surgery (months)	Median 3.0 [range 0–22]				
≤ 3	42 (35)	10 (37)	14 (37)	17 (33)	n.s.
> 3–6	35 (30)	9 (33)	10 (26)	17 (33)	
6–9	25 (22)	5 (19)	8 (21)	12 (23)	
> 9	15 (13)	3 (11)	6 (16)	6 (11)	
Months from surgery to testing (months)	Median 16 [range 6–39]				
6–12	28 (33)	3 (11)	13 (34)	12 (23)	n.s.
12–24	58 (42)	18 (67)	15 (40)	25 (48)	
> 24	31 (25)	6 (22)	10 (26)	15 (29)	

Values presented are mean ± standard deviation or the number (%) of athletes unless otherwise stated. Level of football was divided based on the division of Swedish football the athletes played. Elite was considered the top two divisions, sub-elite the third and fourth divisions, and recreational the fifth division and youth football

^a^Meniscal injury could include injuries to either the medial, lateral, or both menisci

There were no differences in tuck jump score based on duration of rehabilitation (n.s.), nor were there any differences in the proportion of athletes who had abnormal tuck jump scores (n.s.) (Table 2).

**Table 2:** Tuck jump score and duration of rehabilitation

Duration of rehabilitation (months)	Tuck jump score (mean ± standard deviation)	Number of athletes with an abnormal tuck jump score (%)
< 6	5 ± 2	14 of 27 (52)
6–9	5 ± 2	13 of 38 (34)
> 9	5 ± 2	23 of 52 (44)

An abnormal tuck jump score was a total tuck jump score ≥ 6 [10, 20]

**Fig. 2:**
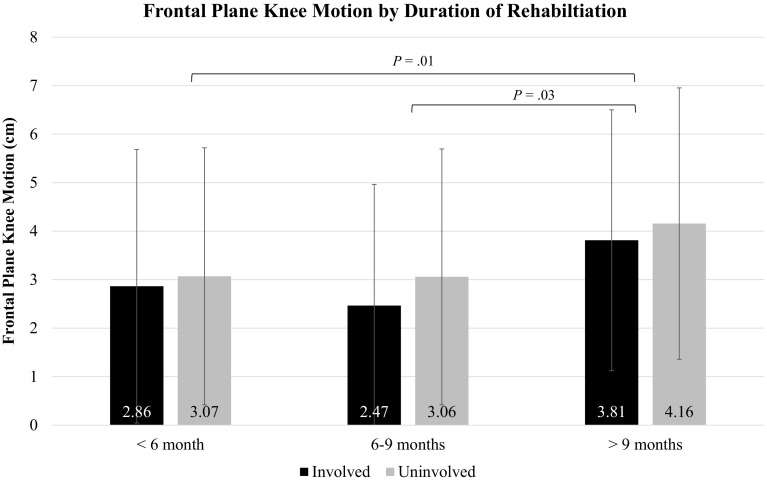
Frontal plane knee motion by duration of rehabilitation. Positive frontal plane knee motion values indicate that from initial contact to the end of the deceleration phase of the DVJ landing the knees move medially into a valgus position. There was no limb × duration of rehabilitation interaction effect (n.s.), or main effect of limb (n.s.); however, there was a significant main effect for duration of rehabilitation (*P* = 0.01). *P* values on the graph represent the significant differences between durations of rehabilitation found during planned comparisons

There was no limb × duration of rehabilitation interaction effect for frontal plane knee motion (n.s.), nor was there a main effect of limb (n.s.) indicating no significant asymmetry during the DVJ. However, there was a significant main effect of duration of rehabilitation [*F*(2,113) = 4.92, *P* = 0.01, *np*^2^ = 0.08], indicating that regardless of limb there was a difference in frontal plane knee motion based on duration of rehabilitation (Fig. 2). Planned comparisons indicated that athletes who had < 6 months (*P* = 0.01) or 6–9 months (*P* = 0.03) of rehabilitation had less frontal plane knee motion during the DVJ than an athlete who had > 9 months of rehabilitation. There was neither limb x duration of rehabilitation interaction effect (n.s.) for pKAM during the DVJ, nor main effects of limb (n.s.) or duration of rehabilitation (n.s.) (Table 3).

**Table 3:** Probability of high peak knee abduction moment (pKAM) by limb and duration of rehabilitation

Duration of rehabilitation (months)	Probability of high peak knee abduction moment (%)	
	Involved limb	Uninvolved limb
< 6	60 ± 27	62 ± 23
6–9	59 ± 25	63 ± 23
> 9	67 ± 24	71 ± 22

## Discussion

The most important finding of this study was that tuck jump score did not differ based on duration of rehabilitation after ACL reconstruction. The women had no difference between limbs or in probability of a high peak knee abduction moment during DVJ landing based on duration of rehabilitation. There was also no difference between limbs in frontal plane knee motion. However, regardless of limb, athletes with > 9 months rehabilitation had more frontal plane knee motion than those with < 9 months. Although increased medial/valgus knee motion has been associated with a higher risk of both primary and secondary ACL injuries [14, 15, 26], with no minimal clinically important difference values it is not possible to determine if the differences between rehabilitation durations are clinically meaningful. Regardless, the implications of this study are that time or duration of rehabilitation alone cannot be used to determine when an athlete is ready to be discharged from physiotherapy or return to play.

A systematic review by Barber-Westin et al. [3] showed that time from surgery was the most common criteria for return to sport clearance. 72% of the included studies had no criteria or used time alone to determine an athlete’s readiness to return to sport [3]. The results of the present study warn against using only time. This study did not collect the details (exercises, progressions, compliance, or complications) of each athlete’s rehabilitation or outcome measures upon discharge; however, it is possible that athletes who received > 9 months of rehabilitation did so because they had difficulty resolving impairments or had complications during rehabilitation. Slower courses of rehabilitation could be caused by a number of factors such as the presence of concomitant meniscal or cartilage pathology (particularly if surgically managed), conservative surgeon or rehabilitation protocols, difficulty achieving range of motion goals, quadriceps inhibition or atrophy, poor neuromuscular control, low motivation, issues with compliance, or difficult psychosocial situations. The presence of concomitant injuries was controlled for in the analysis, but it is still possible that these other factors lengthening rehabilitation could also lead to poorer outcomes with regard to knee control during jump landings. In contrast, shorter periods of rehabilitation could represent athletes who had good neuromuscular control and were able to quickly resolve all impairments, achieve goals and outcome-based milestones. Regardless, the results undermine the idea that a longer duration of rehabilitation necessarily leads to better outcomes. Time alone does not always accurately represent volume of rehabilitation an athlete receives or their compliance. More research is needed into why athletes in this study with longer durations of rehabilitation had more of frontal plane knee motion. However, the more important implication of this study is that purely using time alone is insufficient for guiding rehabilitation, particularly discharge and return to sport.

A community-level study found that athletes who had ≥ 6 months of rehabilitation with structured agility and plyometric training achieved ≥ 90% quadriceps strength and single-legged hop test limb symmetry [7], whereas athletes who had < 6 months of rehabilitation had limb symmetry indices under 85% [7]. The present study set out to examine if the same relationship, longer duration of rehabilitation relating to more symmetrical performance, extended to DVJ landing performance, a higher level more sport-like task. There were no differences between limbs for either frontal plane knee motion or the probability of high peak knee abduction moment. It is possible that a longer duration of rehabilitation is related to symmetry in lower level tasks, but not higher level jump landing tasks. Or differences in the findings of the two studies could be due to how rehabilitation duration was categorized, follow-up time point, or the choice to use bilateral as opposed to unilateral jumping tasks. However, the source of differences really does not matter, as the more important implication is the necessity of objective measures to guide rehabilitation, rather than time.

Future studies are needed to examine rehabilitation prior to ACL reconstruction and the interaction between rehabilitation before and after surgery. Details of rehabilitation protocols, interventions, rehabilitation progression guidelines, dosage, compliance, and return to sport criteria were not collected in this study. Rehabilitation details would have enabled the authors to understand the results in further depth, however support future work on the outcomes of specific interventions and guidelines for progression during ACL reconstruction rehabilitation.

There are some limitations to this study. Although three-dimensional motion analysis allows for more precise measurements and force plates facilitate the calculation of peak knee abduction moment, the two-dimensional video used in this study is clinically accessible and feasible, and both the DVJ and tuck jump are tests used frequently to assess jump landing performance [13, 19, 20]. This is important clinically as both tests can be used to provide the athlete with feedback on their technique and the clinician with specific cues or targets. The tests can be used to assess progression through later stages of rehabilitation, as athletes are performing more advanced sport-related tasks, or as part of return to sport testing [18, 23].

The primary clinical take-home from the current study is the importance of using objective criteria to assess athletes as they progress through their rehabilitation after ACL reconstruction. Objective criteria should include functional measures such as strength and patient-reported outcome measures [7, 11], but as per this study likely also involve higher level sport-related movements. This study indicates that using time alone as a return to sport criteria is insufficient, as there may be differences in movement patterns based on an athlete’s duration of rehabilitation.

## Conclusions

This study found no differences in tuck jump score or probability of high peak knee abduction moment during DVJ landing. Regardless of limb, female football players who had > 9 months of rehabilitation had more frontal plane knee motion during DVJ landing than athletes with < 9 months of rehabilitation after ACL reconstruction. In conclusion, these results suggest that return to sport should not be determined purely based on time or duration of rehabilitation, but rather reinforce the need to use objective measures to guide rehabilitation progression and discharge.

## Abbreviations

ACL: Anterior cruciate ligament
DVJ: Drop vertical jump
pKAM: Probability of a high peak knee abduction moment
*np*^2^: Partial eta squared
